# Cardiovascular Inflammation 2012: Reactive Oxygen Species, SUMOylation, and Biomarkers in Cardiovascular Inflammation

**DOI:** 10.1155/2013/953463

**Published:** 2013-03-10

**Authors:** Jun-Ichi Abe, Ichiro Manabe, Masanori Aikawa, Elena Aikawa

**Affiliations:** ^1^Department of Medicine, University of Rochester Medical Center, Rochester, NY 14586, USA; ^2^Department of Cardiovascular Medicine, Graduate School of Medicine, The University of Tokyo, Tokyo 113-8655, Japan; ^3^Cardiovascular Medicine, Brigham and Women's Hospital, Harvard Medical School, Boston, MA 02115, USA

Cardiovascular diseases are growing burden in westernized countries. Cardiovascular diseases, including atherosclerosis, associate with chronic inflammation. The goal of this special issue is to highlight the inflammatory nature of cardiovascular diseases and their association with the production of cytokines, reactive oxygen species (ROS), and SUMOylation in atherosclerotic plaques. In addition, biomarkers that reflect the biological processes of progression and destabilization of atherosclerotic plaques would provide noninvasive means to identify high-risk patients for cardiovascular events. Armed with this understanding, it is hoped that novel therapeutic strategies will aid in the prevention and management of chronic cardiovascular inflammation and benefit the patients afflicted with cardiovascular disease.

Increasing pieces of evidence support the important role of ROS and cytokine production in the process of atherosclerosis via regulating various signaling pathways leading to vascular inflammation. In the present review, N. T. Le et al. focused on ROS-mediated SUMOylation, which is one of the posttranslational modifications, and discussed its possible implications on vascular inflammation. It becomes apparent that ROS production can regulate the process of SUMOylation in both vasculature and heart and mediate a number of biological processes, such as apoptosis and inflammation. The crucial role especially of ERK5, p53, and MK2 SUMOylation in the process of endothelial inflammation has been discussed. Furthermore, a recent study from their group has found a strong correlation between C_247_T SNP of the NADPH oxidase p22phox subunit and cardiovascular events in postinfarction patients with concurrently high levels of HDL cholesterol and CRP. These data suggest that ROS not only affects the intracellular signaling but also can produce dysfunctional HDL. Since ROS can increase nitration and chlorination of specific tyrosine residues on apolipoproteinA-I, which is a major apolipoprotein component of HDL particles and major mediator of HDL functionalities, it is intriguing to assume that C_247_T SNP of the NADPH oxidase p22phox subunit relates to this HDL modification, leading to dysfunctional HDL. 

R. N. England and M. V. Autieri discussed the role of novel and undercharacterized cytokine IL-19 on vascular inflammation. IL-19 shows pleotropic effects on vascular wall. It can inhibit proliferative and proinflammatory gene expression in vascular smooth muscle cells (VSMCs) via NF-kB independent signaling, which is very different from the effects of IL-10. The possible involvement of the expression of suppressor of cytokine signaling 5 (SOCS5), human R antigen (HuR), and hem oxygenase-1 (HO-1) in this inhibitory effect has been discussed. In contrast to VSMCs, IL-19 showed proliferative, promigratory, and proangiogenic effects in vascular endothelial cells (ECs), although its role in EC inflammation remains unclear. Since IL-19 can inhibit ROS production and atherosclerosis formation in a mouse model, investigation the exact mechanisms of and interplay between IL-19 and ROS in both VSMCs and ECs will be valuable for orchestrating anti-inflammatory therapies for cardiovascular events. 

Biomarkers can reflect the biological processes of disease progression. For instance, CRP levels have been shown to be predictive of cardiovascular risk. However, CRP is a nonspecific inflammatory marker that is affected by any inflammatory conditions. Therefore, it is important to develop biomarkers that specifically report the biological processes in atherosclerosis and other cardiovascular diseases, associated with chronic inflammation. N. Kafkas et al. showed that serum neutrophil gelatinase-associated lipocalin (NGAL) levels were increased in patients with coronary artery disease. They propose that NGAL could be used to discriminate patients with acute coronary syndrome from those who are with stable angina or without coronary artery diseases. V. Panichi et al. review newer biomarkers, including CD40 ligand and pentraxin-3 that reflect chronic inflammation in cardiovascular and renal diseases. D. Vasic and D. Walcher summarize the current knowledge about C-peptide as a biomarker and bioactive molecule. The deposition of C-peptide may promote inflammation.

The docking proteins of the Grb2-associated binder family (Gab1, Gab2, and Gab3) serve as important signaling compartments. They may amplify and integrate signal transduction pathways in response to various stimuli such as cytokines. Interestingly, recent evidence has linked abnormal Gab signaling with human diseases including cancer and cardiovascular disease, for which inflammation plays a key role. Y. Nakaoka and I. Komuro. extensively described molecular structure, recruitment, and phosphorylation of Gab proteins and discussed their role in cardiovascular and cancer inflammation.

An emerging concept suggests that chronic inflammation participates in the pathogenesis of atherosclerosis and other cardiovascular diseases. Thus, controlling proinflammatory pathways such as ROS-mediated SUMOylation may attenuate such diseases. The goal of this thematic series is to highlight emerging proinflammatory mechanisms involved in cardiovascular inflammation. We believe that this issue will offer updated concepts and help readers develop ideas leading to future investigations and new drug development.

*Jun-Ichi Abe* *Jun-Ichi Abe* *Ichiro Manabe* *Ichiro Manabe* *Masanori Aikawa* *Masanori Aikawa* *Elena Aikawa* *Elena Aikawa*

